# Decision-making for children and adolescents: a scoping review of interventions increasing participation in decision-making

**DOI:** 10.1038/s41390-024-03509-5

**Published:** 2024-10-06

**Authors:** Inga Bosch, Hermann Siebel, Maike Heiser, Laura Inhestern

**Affiliations:** Department of Medical Psychology, University Medical Center Hamburg-Eppendorf, Hamburg, Germany

## Abstract

**Purpose:**

To review and synthesize the literature on interventions to facilitate shared decision-making or to increase participation in decision-making in pediatrics focusing on interventions for children and adolescents.

**Methods:**

We systematically searched three electronic databases (September 2021, update in September 2022). We included studies that aimed to increase involvement of children and adolescents in medical or treatment decisions, regardless of study design and reported outcomes. Study quality was assessed using the MMAT. The synthesis strategy followed a narrative methodology.

**Results:**

21 studies met the inclusion criteria. Interventions aimed to increase participation by provision of information, encouraging active participation and collaboration. Didactic strategies included digital interactive applications (*n* = 12), treatment protocols and guiding questions (*n* = 12), questionnaires or quizzes about patients’ condition or their knowledge (*n* = 8), visual aids (*n* = 4), and educational courses (n = 1). Findings indicate positive effects on some of the investigated outcomes. However, the heterogeneity of studies made it difficult to draw consistent conclusions about the effectiveness of interventions.

**Conclusions:**

Interventions used a variety of approaches to facilitate SDM and increase participation. The findings suggest that interventions have inconsistent effects across different outcome variables. The evidence was limited due to the methodological shortcomings of the included studies.

**Impact:**

- To increase the participation of children and adolescents in decision-making, interventions targeting them are needed. Most intervention focus on the provision of information and encouragement for active participation.
- The results suggest high feasibility and, mostly, positive effects in participation, health-related knowledge, patient-HCP relationship, and adherence
- The study highlights that further high-quality studies using similar outcome parameters are needed to investigate the effects of interventions to facilitate participation in decision-making.

## Introduction

In pediatrics, decision-making does not only include the patient and the healthcare professional (HCP), but also the parents as surrogated for a child’s decision. Hence, the process of decision-making becomes triadic. It has been shown that interventions to support decision-making in pediatrics mainly target parents^1^ and that children and adolescents are often not involved in treatment or decision processes or that their wishes are not taken into account.^2–4^

Preferences of children and adolescents show, that they want to be addressed directly in healthcare contexts and want to play an active role in issues related to their health and treatment.^5,6^ A recent study shows that almost three out of four adolescents prefer active participation and shared decision-making (SDM) in medical decisions.^7^ Most adolescents want to be at the center of their medical encounter and to be treated as adult patient,^8^ whereas some children and adolescents experience high pressure and distress when being involved in decision-making.^9,10^ Therefore, healthcare encounters need to take into account child’s and adolescent’s preference for participation.^11–13^ To identify patients’ preferences of involvement, information and communication provided by HCPs should be age-appropriate and proactively targeted at children and adolescents.^14^ In addition, HCPs should enable children and adolescents to participate in their healthcare by providing information about the risks and benefits of decisions, giving them the opportunity to interact with experts, and allowing them to reflect on their own values. Adolescents have been shown to have competencies relevant to medical decision-making.^15^ Active participation in medical decisions can lead to reduced anxiety and increased sense of control in children and adolescents.^16^ In addition, adherence to treatment and self-management of illness and health are positively associated with participation.^17,18^

While the development and implementation of interventions to facilitate shared decision-making (SDM) in adult care have been promoted in recent decades,^19,20^ recent studies suggest that routine implementation of SDM interventions in pediatric healthcare is rare.^21,22^ However, there is an increasing awareness for the participation of children and adolescents in decision-making processes.^23^ Interventions have been developed to facilitate participation.^1,24,25^ Many of these interventions focus on parents as relevant partner in pediatric medical decision-making.^1^ Interventions targeting children or adolescents or focusing on HCP-child dyads are limited.^26–29^ Nevertheless, information and evidence on decision-making interventions in pediatrics may provide relevant information for HCPs to promote patient participation, satisfaction, and knowledge and to enhance health outcomes and treatment adherence.^25^ According to the classification suggested by Grande et al.,^30^ different types of tools can support and facilitate the decision-making process.^30^

Our aim was to provide an overview of interventions that increase participation in decision-making (including SDM interventions) that target at children and adolescents or HCP-child dyads. Our focus was to systematically describe the content and didactic strategies of these interventions. We also aimed to review findings on the effectiveness of interventions.

## Methods

We conducted a scoping review using a systematic literature search to follow our study aim. The reporting of this scoping review follows the Preferred Reporting Items for Systematic Reviews and Meta-Analyses (PRISMA)-Statement extension for Scoping Reviews (Supplementary Material S1).

### Search strategy

Search terms were defined according to the PICO criteria.^31^ For each aspect of PICO, relevant search terms were gathered and applied in our systematic search. The final search included terms related to decision-making, patient participation, pediatrics, and healthcare. Full search strategies are provided in Supplementary Material S2. We searched the following electronic databases in September 2021: CINAHL, PsycINFO, and PubMed. In addition, we performed a systematic reference and citation screening of the retrieved full texts and related systematic reviews. The search was updated in September 2022.

### Eligibility criteria

#### Population

We included studies of decision-making interventions for children and adolescents (up to 18 years). If young adults were included in a study, the intervention also had to be aimed at children and/or adolescents and they had to be included in the study. As the focus of the scoping review was to obtain an overview of interventions, it was not relevant whether the results were reported separately for children, adolescents, and young adults. There was no restriction to a specific disease or treatment. Studies were excluded if the interventions focused on parental decision-making or if the HCPs were not working in pediatric setting (Table 1).

**Table 1:** Inclusion and exclusion criteria based on PICOS framework^31^.

	Included	Excluded
Participants	Children 18 years of age or younger with medical condition If young adults were included in a study, the intervention also had to be aimed at children and/or adolescents and they had to be included in the study. As the focus of the scoping review was to obtain an overview of interventions, it was not relevant whether the results were reported separately for children, adolescents, and young adults.	Adult patients (19 years of age or older) Healthcare providers Parents only
Intervention	Intervention focusing on SDM or increasing participation in the decision-making process in children and adolescents in the pediatric health context (e.g., treatment decision) except excluded settings	No SDM Intervention or no intervention to increase participation in decision-making process, intervention focusing on parents’ decision-making for their child; Interventions on hypothetical decisions, decisions in non-clinical settings (e.g., school), decisions about pregnancy, peri- or neonatal care, decisions on participation in research, decisions on advanced care planning; Interventions focusing on transition; Self-management interventions;
Comparison	All comparison groups, including none	
Outcomes	Description of the intervention Feasibility or usability of the intervention Effects of the intervention	Presentation of only the development and design of the intervention, Missing or insufficient description of the intervention, no data on usability, feasibility or effectiveness
Study methods	All study designs with original data	Reviews
Other	Peer-reviewed articles	Commentaries Unpublished studies Grey literature No peer review

#### Intervention

Interventions were included if they aimed to increase patient involvement in decision-making (information, activation, and/or collaboration) or to facilitate SDM. There were no restrictions on the setting or format of the interventions. Studies were excluded if they focused on hypothetical decisions, non-medical decisions (e.g., school), decisions about pregnancy, peri- or neonatal care, advanced care planning, decisions about participation in research, or self-management interventions without a focus on decision-making.

#### Outcome

In addition to information and details about the interventions, any measures of the usability, feasibility, or effectiveness of the interventions were included. Studies were excluded if (a) they only presented the development and design of the intervention, (b) the description of the intervention was missing or not sufficiently reported, and/or (c) no data on usability, feasibility, or effectiveness were provided.

#### Study design

We included all types of studies that reported original data on the feasibility or effects of the interventions (qualitative, quantitative, or mixed-methods design). Studies were excluded if they were literature reviews, commentaries, or unpublished research.

Other inclusion criteria were publication in a peer-reviewed journal and German, English, or Spanish language.

### Study selection

Studies identified by the search strategy were exported from the databases to Endnote software. Duplicates were identified and removed. Title and abstract screening was performed according to the inclusion and exclusion criteria. Approximately 50% of the title and abstract screening was done by two of the authors (IB, HS). Full-text screening of the identified relevant articles and search of citations and references was performed independently by two reviewers (IB, HS or LI). In case of ambiguity regarding inclusion, eligibility was resolved by discussion with LI.

### Data extraction and quality assessment

Data extraction followed predefined variables covering citation, country, target group, and description of intervention, study design, outcome parameters, and study outcome.

Quality assessment was performed for studies that included any type of evaluation of their intervention. The methodological quality of the included studies was assessed by the first author (IB) and double-checked by one of the other authors (HS, MH) using the Mixed Methods Appraisal Tool (MMAT).^32^ Disagreement was resolved with LI. The MMAT allows for the quality assessment of studies with different study designs and has been shown to be reliable and valid.^32^

### Data synthesis

The synthesis strategy followed a narrative methodology. Study characteristics and results were integrated and summarized. Interventions were classified as a) information, b) information and activation or c) information, activation, and collaboration.^30^ A meta-analytic strategy was not feasible due to heterogeneous study designs and outcome parameters.

## Results

### Study selection

**Fig. 1:**
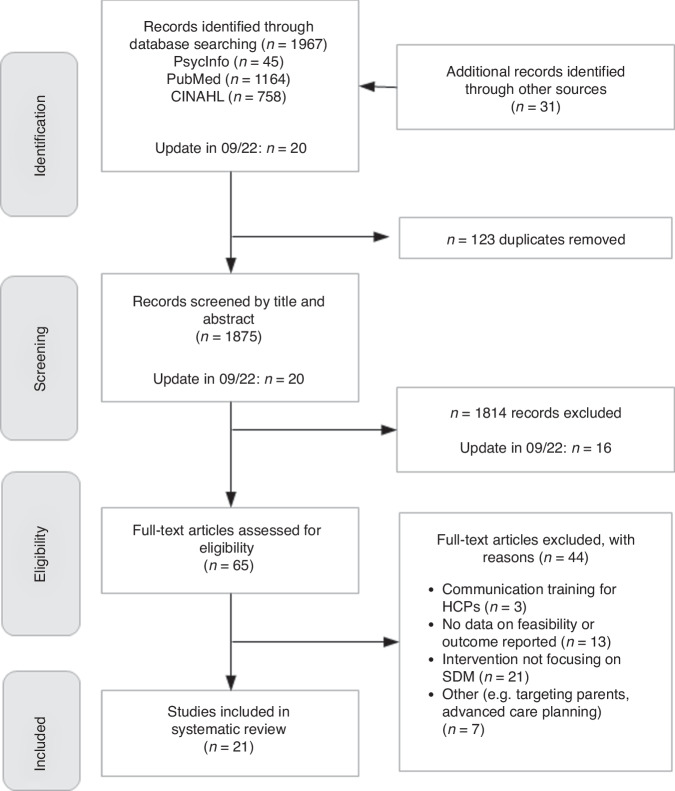
Flow chart of study selection.

A total of 1967 studies were identified through our systematic electronic search and 32 additional articles were identified through other sources. The search update in September 2022 yielded a further 20 articles. After removing duplicates, 1899 titles and abstracts were screened for eligibility. In the end, 65 full texts were retrieved. Of these, 44 were excluded for various reasons, leaving a final sample of 21 for the synthesis (Fig. 1 and Supplementary Material Table S2).

### Study characteristics

A detailed overview of all included studies, including descriptions of the interventions and their results, is provided in Table 2 (see also Supplementary Material S3 for references of Table 2).

**Table 2:** Characteristics and results of included studies and interventions

Author (Year, Country)	• Type of intervention^a^ •Target group	Aim/Intention	Content of the intervention	1) Evaluation/Design 2) Measurement of results^b^	Results
Carlsson et al. (2021, Sweden)	1) Information and Activation using a digital, interactive communication tool “SISOM“ 2) Children with different diseases	To Record children’s situation and symptoms, basis for dialog with professionals;	Application on mobile device. Children design an avatar and go on a virtual journey through five islands (dealing with things, my body, my thoughts and feelings, things to be afraid of, in the hospital). 84 animated questions to describe the children’s life situation and symptoms. Selection of severity on 5-point Likert scale with cartoon faces (different colored smileys). Resulting summary is printed for child and HCP	1) Qualitative: Unstructured interviews with children (*n* = 16) 2) Thematic content analysis	A communication space was created. Child was included in communication, recognized as a person and time was given. Identified themes of the children: • Enticing me to speak • Avoiding speaking, but still being heard • Making me think
Eldbrooke-Childs et al. (2019, England)	1). Information and Activation using the digital Application “Power Up” 2). Children and adolescents with mental health problems	To Empower young people to take an active role in decisions that impact their health and care	Power Up enables young people to record their questions, plans, decisions, and diary entries and supports young people to identify individuals in their support network with whom they would like to share these entries. It provides a digital space for young people to prepare what they want to bring to conversations about their mental health and well-being, including the following main features: (1) My People: At the center of Power Up, young people can add people in their support network. Users can flag information entered in other sections of the app to specific people in their support network, and all content flagged for sharing with a specific person from other areas of the app is displayed in My People. If an entry is not flagged to My People, it is stored chronologically; otherwise, a young person can prioritize which entries should appear first. (2) My Diary: A space for users to express what is going on for them in their daily lives. (3) My Plans: A section devoted to adding all plans and goals, including what to do in specific circumstances related to users’ mental health, such as anxiety-provoking situations. (4) My Questions: Young people can enter any questions they have or wish to discuss with carers, friends, teachers, or clinicians and keep a record of the answer after it has been discussed. (5) My Decisions: A space for young people to work through decisions, weighing up the pros and cons associated with decisions using a visual weighted scale. (6) There is the option for all entries in the above sections to be inputted in the form of photo, video, audio, or text. (7) Entries such as photos or phone numbers can only be viewed within Power Up, not in the phone’s main library or phonebook. (8) Help and Support: A selection of resources that give the young person a series of links to websites and phone numbers. There are a set of prestored resources, however, the young person can also add his or her own. These can be called or visited directly from the app. (9) Power Up is secure and password protected.	1) Qualitative: Short semi-structured interviews about acceptability, only with participants from IGs (specialist strand: *n* = 6; schools strand: *n* = 5) 2) Thematic content analysis	Usage data showed that there were an estimated 50 (out of 64) users of Power Up in the intervention arms. The findings of this study indicate that the app is acceptable. Three main themes • Motivation for use: Different motivations were outlined, the ability to make entries into the app via multiple modalities was evaluated positively by young people. They also expressed the need to be able to trust that the technology was a private platform, which could not be accessed by others, unless users chose to share it with them. The app aided young people to remember important things to either share in a consultation with a HCP, or to reflect on their thoughts over time. Power Up was also sought by participants when they could not speak to anyone else about their concerns and emotional experiences. Finally, the accessibility of Power Up in the moment was a key motivating factor for young people’s engagement with the technology. • Impact of use: Ranged from allowing young people to derive new insights from documenting their experiences, through to encouraging conversation with others in their support network. Young people explained how using the app had allowed them to see changes in their emotional state over time, marking progression in their psychological journey. It helped them to gain greater understanding about themselves, as well as to clarify their thinking by weighing up the pros and cons of significant issues in a balanced, considered way. The app also mediated communication with important people in the participants’ support network • Barriers to use: Technological difficulties, disengaging from the app because of experiencing difficulties in their personal circumstance. Power Up had not been fully embedded into mental health services and was seldom incorporated into clinical sessions between young people and their therapists
El Miedany et al. (2019, Egypt)	1). Information and Activation using the Visual, interactive decision-making aid 2). Children and adolescents with idiopathic arthritis	To support the decision-making ability of patients	Printed and electronic version of decision aid used prior to clinical encounter by patients themselves. Specialized nurse is available to answer questions. Instrument informs children about treatment options, side effects, medication use, and asks about patient’s goals and values.	1). Quantitative: Randomized controlled trial with posttest (*n* = 220) a. Perceived SDM: 9-item Shared Decision Making-Questionnaire (SDM-Q-9; Kriston et al., 2010) b. Therapy adherence c. School absenteeism d. Quality of life	a. 70% of IG (vs. 30% CG) agreed that HCPs wanted to know how they would be involved in decision-making (*p* < 0.01). 88% of the IG (vs. 38% of the CG) felt involved in the selection of options (*p* < 0.01). 89% of IG (vs. 41% CG) had agreed with HCP on further treatment (*p* < 0.01). b. 88% of IG (vs. 71% of CG) adhered to their drug therapy (*p* < 0.01). In the IG, the likelihood of discontinuing medication due to intolerance was lower. c. School absenteeism was significantly lower in the IG (*p* < 0.01) d. Reported quality of life was significantly higher in the IG (*p* < 0.01): children reported fewer worries about the future and were better able to cope with everyday activities
Gilljam et al. (2020, Norway)	1). Information and Activation using a digital, interactive communication tool “SISOM“ 2). Children with cancer	Record children’s situation and symptoms, basis for dialog with professionals	Application on mobile device. Children design an avatar and go on a virtual journey through five islands (dealing with things, my body, my thoughts and feelings, things to be afraid of, in the hospital). 84 animated questions to describe the children’s life situation and symptoms. Selection of severity on 5-point Likert scale with cartoon faces (different colored smileys). Resulting summary is printed for child and HCP	1). Mixed-methods: Quasi-experimental design with parallel-mixed method to combine qualitative and quantitative data (*n* = 27) 2). Video recordings of routine appointments Quantitative: a. Time of the children speaking and the number of expressions by the HCPS addressed to the children b. Observation scheme to evaluate the child’s verbality (verbally inactive, limited verbal, moderately verbal, verbally active) c. Level of participation (Shier, 2001): The children were listened to; Children were supported in expressing their opinion; The views of the children were taken into account; Children had the opportunity to participate in the decision-making process; Responsibility of HCPs Qualitative: d. Observed episodes of participation: focused, deductive content analysis	a. Larger proportion of HCP’s expressions were directly addressed to the child in the IG (71% vs. 57% in CG). Children spoke to HCPs for an average of 1.1 min in the IG vs. 1.8 min in the CG b. Verbality of children in IG was slightly higher than in CG. c. Higher score for children’s participation in the use of “SISOM” compared to the CG, especially in the three lower levels. d. More observed episodes in first two levels in IG compared to CG (level 1: 21 vs. 5, level 2: 100 vs. 32). No differences in Level 3. Levels 4 and 5 not evident in any appointment. New category that is not in Shier’s model emerged: children receiving information. Of these, more observed episodes in IG than CG (84 vs. 36).
Ho et al. (2021, Canada)	1. Information and Acivation using a Patient decision aid (PtDA) 2. Children/Adolescents (8–19 years) with elbow flexion contracture as part of brachial plexus birth injury (BPBI)	Develop a PtDA following International Patient Decision Aid Standards (IPDAS) and use this PtDA prototype to help adolescents and their families with treatment-related SDM	(1) Information about nerve injuries, including section “What Kids Want to Know” (2) Information about elbow flexion contractures (3) Information about possible treatment options for elbow flexion contractures which have to be decided between using the present PtDA a. Watch and Wait b. Stretching Program (Cast and Splint) c. Elbow Release Surgery (4) Make A Decision About Treatment including a quiz on knowledge of key facts about treatment for elbow flexion contracture as well as sections “How comfortable are you about making a decision?” and “Are you ready to make a decision?” (5) Decision Support Tools: a. Worksheet where patients can list the things that matter most about having either treatment and compare pros and cons b. VCM questionnaire with questions that help to think about how patients feel about treatment options; sum score can point in either of the 3 possible treatments and thus help with decision (6) Support: Who else is involved in this decision?, including Support Worksheet with space to write down who else is involved, what the other person would decide and how the patient feels toward this (i.e., pressured) (7) Testimonials from families with experience with either of the treatment options	1) Mixed-methods quantitative: Uncontrolled design using questionnaires to quantitively field-test the developed PtDA (*n* = 17 adolescents) Qualitative: In-depth recorded interviews (*n* = 5 adults; 14 children/adolescents; 13 parental units) and unstructured participant observation in clinic setting (*n* = 15 families with children from 5 to 16; 19 HCPs) to identify important themes and develop the present PtDA; Semi-structured cognitive interviews with anticipated probes to filed-test PtDA (*n* = 17 adolescents) 2) Quantitative: a. Overall comprehensibility of PtDA prototype: Flesch Kincaid Grade Level Readability Test (Kincaid et al., 1975) b. Sensibility and feasibility of PtDA prototype: 8-item questionnaire with 6 close-ended and 2 open-ended questions Qualitative: c. Identifying themes and content for PtDA: In-depth interview with inductive coding (Leech & Onwuegbuzie, 2007) followed by directed content analysis (Hsieh & Shannon, 2005) d. Enriching overall qualitative analysis: Assessing field notes taken during participant observation of families and clinicians in clinic setting e. Comprehension, Retrieval, Confidence & Response regarding use of the developed PtDA: Directed content analysis (Hsieh & Shannon, 2005) of cognitive interviews	a. Flesch Kincaid Grade Level of PtDA prototype was 6.2 which reveals desirable readability b. Descriptive data (questionnaire scores) show that a majority of participants found the PtDA prototype to be clear and helpful (M = 75.1), that the amount of time (M = 65.3) and information (M = 74.2) was just right or said that they will use the PtDA for their own decision (M = 84.4). Of the 7 participants who commented on what they did not like about the PtDA, 4 felt that some concepts needed clarification, while 2 disliked the ‘amount of reading’ and felt that the PtDA was too long c. 9 identified themes used to develop the PtDA prototype: Clinic Factors That Influence Family Decision Making, Treatment Options, Treatment Considerations, Youth/Family Education, Parental Decision Making, Youth Decision Making, BPBI and Elbow Flexion Contracture, Adjustment to BPBI and Effect on Decision-Making, PtDA Format and Content d. 9 identified areas in which problems emerged during use of PtDA prototype: Comprehension – medical terms; Comprehension – concepts; Communication; Face validity; Retrieval; Visual aids; Actionability; Content; Emotional response
Hulin et al. (2017, England)	1. Information and Activation using a dental Decision Aid guided by the Ottawa Personal Decision Guide 2. Children/Adolescents (10–16) years and their parents	Development and field-testing of a Decision Aid Booklet in dental care for children faced with the decision to undergo dental treatment with inhalation sedation, intravenous sedation, or general anesthesia	a) Introduction: Description of the condition relating to the healthcare decision, the decision being made, the options available to the patients b) Information: List of the positive and negative features of the options available to the patient side by side in a question-and-answer table c) SDM: An explicit values clarification exercise and a short multiple-choice quiz relating to the treatment options available	1. Mixed-methods Qualitative: Interviews with children/adolescents (*n* = 12), parents (*n* = 13); Expert clinician focus grouo (*n* = 6) and expert patient group interviews (patient *n* = 3, parents *n* = 3) Quantitative: Quasi-experimental design, CG patients and parents given treatment as usual, IG given treatment as usual plus the Decision Aid Qualitative: Thematic content analysis Quantitative: a) Decisional conflict: Decisional Conflict Scale (Stacey et al., 2014) b) Knowledge increase: Adapted Scale from the knowledge questionnaire provided through the Ottawa Decision Support Framework (O’Connor, 2012) c) Dental anxiety: Modified Child Dental Anxiety Scale (Wong et al., 1998)	a.) No significant differences in overall decisional conflict or on any of the subscales between CG and IG for both patients and parents b.) Knowledge scores were significantly greater for patients (*p* = 0.01) and parents (*p* = 0.03) in the IG in comparison with patients in the CG c.) Dental anxiety did not significantly differ between IG and CG
Iio et al. (2022, Japan)	1. Information and Activation using a prototype mobile asthma app based on results of previous survey 2. Different app contents for different age groups: Children aged 0–6 years with asthma and their HCPs and children aged 7–12 years with asthma and their HCPs	Increase feelings of self-efficacy in asthma management, improve asthma knowledge and facilitate continuous self-management behavior of controlling asthma symptoms	Common app contents and functions for both age groups: Self-monitoring of medications and symptoms, preparing for asthma exacerbation and disasters, asthma action plan, tailored feedback according to the Japanese pediatric asthma control test, medication alert, app input alert, notification of the results in pediatric asthma control test. Individualized feedback messages based on medication usage are displayed for HCPs App contents for children aged 0–6 years and their HCPs: Focus on asthma knowledge via pictorial book about condition, causal factors, and complicating factors with a quiz in the end. Mainly for HCPs, but book could be completed by children with help from HCP. Book also promotes interaction between child and HCP App contents for children aged 7–12 years and their HCPs: Focus on Asthma knowledge (via manga comics about asthma, medication, and stress management with quiz in the end) and individualized feedback about status of medication usage	1. Mixed-methods quantitative: Non-randomized uncontrolled descriptive study (*n* = 34 pairs of children with asthma and HCPs) Qualitative: Descriptive qualitative research analysis to identify codes, subcategories, and catego ries from the data (*n* = 34 pairs of children with asthma and HCPs) 2. Quantitative: a. Feasibility: 7-item questionnaire according to the feasibility assessment using the 10-statement System Usability Scale (Brooke, 1996) b. App usage: 3-month survey to identify usage frequency of app features; 6-month survey about most used and non-used features; After 6 months: Collection of app data about number of access logs and usage frequency of each feature Qualitative: c. Barriers for continuous use of app and facilitators promoting continuous use of app: 3-month web-based survey questionnaire d. Impressions of app: 6-month web-based survey questionnaire	a. App attributes and percentage of children who agreed: “Good” (63%), “Useful” (45%), “Satisfied” (46%), “Available” (55%), Intention of behavior: “I wanted to act” (46%), “Applicable design” (55%), “Read tailored messages” (64%) b. Most used feature: “record” (calendar/diary and asthma control check), about 61% of children used asthma manga, usage rate of asthma picture book among toddlers (*n* = 12) and their HCPs was 25%; Most used feature: “record”, about 61% of children used asthma manga and asthma quiz, about 80% of participants used asthma control check c. Difficulty in using the app contents was identified in 6 categories: “record”, “preparing”, “alert settings”, “change settings”, “mobile phone owner”, “display and motivation”. HCPs: “It can only enter about asthma” and “It takes time”. Both HCPs and children: “It cannot record my daily physical condition, climate, and events”. Children: “It does not know how to use preparing”, “It cannot display alert”, “It does not know how to use the change settings”, “My mother was typing everything”, “The eggs do not grow and the contents cannot be seen” d. 60% of HCPs and 73% of children showed positive intention of behavior in app usage, HCPs: “It was easy to manage”, “it is aiming for self-management by the child”, “I was too busy to read the tailored messages”, Children: “I did not know where the tailored message was”
Langer et al. (2022, USA)	1. Information, Activation and Collaboration using SDM Protocol for Treatment Planning in Youth Psychotherapy 2. Children/Adolescents (7–15 years) with anxiety or depression as part of adolescent/caregiver dyads	Engage children/adolescents as active collaborators in treatment planning process and thus facilitate the personalization of established evidence-based treatments	1. Introduce SDM: Clinician shares the overall session goal is to work together to design a treatment plan that will work best for the family 2. Practice collaborative decision-making: Clinician describes SDM as a collaborative approach to making decisions with opportunities for patients and clinicians to share their ideas, preferences, and perspectives while considering available evidence 3. Select treatment targets: Clinician summarizes baseline assessment evidence 4. Discuss treatment-related values: Clinician highlights how unique perspectives about treatment characteristics may inform treatment planning 5. Introduce the evidence: Clinician provides basic psychoeducation about the existence of treatment efficacy/effectiveness research in a developmentally appropriate manner 6. Select treatment participants: Clinician presents the research findings related to treatment participants for the youth’s presenting issues and, through discussion, determines who will participate in treatment 7. Select treatment components: Clinician presents the research findings related to treatment components for the youth’s presenting issues 8. Plan symptom tracking and follow-up: Clinician provides information about progress monitoring and introduces a method for tracking progress	1) Quantitative: Randomized controlled clinical trial (*n* = 40 adolescent/caregiver dyads); CG: Clinician-Guided Condition (Clinicians planned treatment alone based on baseline assessment data) 2) a. Feasibility & acceptability of SDM Protocol: Self-developed questionnaire c. Differences in treatment plan components between IG and CG: Treatment session clinical notes, including treatment planning discussions c. Decision-making and related constructs: Decisional Conflict Scale (DCS; O’Connor, 1995), Satisfaction with Decision Scale (SWD; Holmes-Rovner et al., 1996), 11-item Decision Self-Efficacy Scale (O’Connor, 1995), Decisional Regret Scale (DRS; Brehaut et al., 2003), SDM-9 Questionnaire (SDMQ; Kriston et al., 2010), Treatment Outcomes Expectation Scale (TOES), Motivation for Youth Treatment Scale (MYTS; Bickmann et al., 2010), Therapeutic Alliance Scale for Children (TASC; Shirk & Saiz, 1992), d. Diagnoses, symptoms, and functioning: Anxiety Disorders Interview Schedule for DSM-IV, Child/Parent (Silverman & Albano, 1997), Multidimensional Anxiety Scale for Children (MASC; March et al., 1997), Children’s Depression Inventory (CDI; Kovacs, 1992), Clinical Global Impression – Severity and Improvement (CGIS; Guy, 1976)	a. Treatment length, attendance and completion wasn’t significantly different between IG and CG. Caregivers in IG reported significantly greater satisfaction with decisions related to treatment planning (*p* = 0.013) b. Treatment plan components didn’t significantly differ between IG and CG. During treatment, treatment plans were discussed and modified in a significantly higher percentage of sessions in IG (*p* = 0.004) c. IG and CG adolescents didn’t significantly differ on their treatment outcome expectations, decisional conflict, motivation for treatment, and decisional regret. IG adolescents reported significantly lower therapeutic alliance scores directly following the treatment planning session (*p* = 0.017), but not at mid- or post-treatment assessment. They also showed significantly more involvement in the treatment planning process (*p* = 0.037). IG adolescents didn’t significantly change in decision self-efficacy from before to after the SDM session. IG caregivers showed significantly lower decisional conflict (*p* = 0.004), significantly higher satisfaction with the decision (*p* = 0.03), significantly more involvement in treatment-planning process (*p* = 0.014), significantly lower therapeutic alliance directly after SDM session (*p* = 0.03), but not at mid- or post-treatment assessment, and significantly lower decisional regret (*p* = 0.02). Their report of self-efficacy also didn’t change significantly from before to after the SDM session d. No significant post-treatment condition differences between IG and CG
Lawson et al. (2020, Canada)	1) Information and Activation using a decision coaching guided by the Ottawa Family Decision Guide (OFDG), carried out by social workers 2) Children/Adolescents with type 1 diabetes (T1D) as part of adolescent/family dyads or triads	Work through decision-making process together, facilitate decision between treatment options for T1D, encourage SDM among young people with diseases like T1D	One session of decision coaching with social workers following the OFDG OFDG: (1) Clarify your decision including clarifying open-ended questions (2) Explore your decision including a table with space for pros/cons of either option, how much they matter, and a support section asking who else is involved in the decision and how (3) Identify your decision-making needs including close-ended questions (4) Plan the next steps based on your needs including close and open-ended questions asking about knowledge, values and support	1) Quantitative: Uncontrolled pre/post study; T1: baseline, T2: immediately after coaching, T3: 10 to 14 days after coaching (*n* = 45 adolescents) a. Changes of Decisional conflict (T1–T3): Low literacy version of Decisional Conflict scale (DCS; O’Connor, 1995) b. Changes of choice predisposition (T1–T2): Choice Predisposition Scale (O’Connor, 2003) c. Adolescent/parent dyad agreement about preferred choice (T1–T2) d. Satisfaction with intervention (T3): Questionnaire that combines a SDM satisfaction rating tool (Barry et al., 1995) and a modified version of the Genetic Counseling Satisfaction Scale (DeMarco et al., 2004)	a. Adolescents’ total DCS scores dropped significantly from 32 at T1 to 6.6 at T2 (*p* < 0.0001), they were also significantly improved for all subscales, indicating they felt informed, had values clarity, felt supported, and were sure of their decision b. At T1, 33 adolescents were leaning towards insulin pump therapy, 2 tended towards conventional therapy, 1 tended towards multiple daily injections (MDI) and 9 were undecided. At T2, 26 of those first leaning towards an insulin pump still tended towards this option, 2 changed to MDI and 5 were undecided after coaching. 4 of 9 people remained undecided, the other 5 changed to insulin pump at T2 c. From T1 to T2, Agreement about treatment options increased between adolescents and mothers as well as adolescents and fathers and decreased between mothers and fathers d. Of adolescents, 57% rated length of coaching as “just about right”, 8% rated it as “too long”; 92% thought that coaching and OFDG helped them consider their options in a balanced way, 92% thought that the SDM intervention was helpful in aiding them to come to a decision, all participants would recommend coaching and OFDG to others
Lipstein et al. (2022, USA)	1. Information, Activation and Collaboration using a Multi-component SDM intervention 2. HCPs and families with children suffering from pediatric inflammatory bowel disease (IBD)	Support SDM and engage both parents and patients in pediatric IBD decisions	Three-component SDM intervention based on prior research b.) Letter: Sent in advance of the scheduled clinic visit. Supposed to encourage families to prepare for the visit by considering their treatment goals, the impact of IBD on their life, and any concerns. c.) SDM cards: Supposed to facilitate a decision conversation between an HCP and a family. Cards contain simple images and plain language to highlight attributes of treatment choices (e.g., costs, side effects…). HCP invites family to pick the card they want to discuss first and conversation proceeds from there. d.) Follow-up phone call: Designed to facilitate on-going communication between families and HCPs by eliciting questions that might help the family with decision-making and then forwarding those questions to the HCP.	1) Quantitative: Non-randomized controlled (CG: usual care) pre-post pilot study (*n* = 11 HCPs enrolled in both IG and CG; *n* = 18 families in IG; *n* = 18 families in CG) 2) a. Feasibility: Percentage of patients receiving all three intervention components and length of clinic visit, measured as time from HCP entering the room to the final time HCP exits the room (consensus: intervention is considered feasible if 80% of participants received all three components and average clinic visit length did not increase by more than 10% compared to CG) b. Acceptability: Mean score on 5-item survey adapted from other SDM trials (Brinkman et al., 2013; Weymiller et al., 2007) c. Disease activity: 3-month change in disease control score on either pediatric ulcerative colitis activity index (PUCAI; Turner et al., 2007) or short pediatric Crohn’s disease activity index (PCDAI; Shepanski et al., 2003) d. Time to treatment initiation: Number of days from index encounter until patient received first dose of an anti-TNF or prescription for a different treatment e. Adherence: Medication possession ratio (MPR; Steiner et al., 1988) f. Quality of Life: PedsQL generic score (Varni et al., 2003) b. Extent of SDM, decisional conflict and decision regret: OPTION5 questionnaire (Barr et al., 2015) and Shared Decision Making-Questionnaire-9 (SDMQ-9; Kriston et al., 2010)	a. Pre-clinic letter was sent to 17 of 18 families in IG but reported seen only by 5 of 17 families. SDM cards and follow-up call were received by all IG families. Average visit length was 35.5 min in CG and 49.2 min in IG, an average increase of 39% b. Intervention was found to be acceptable by 94.4% of parents and 80% of patients in IG which is not significantly different from results in CG c. Did not significantly differ between CG and IG d. See c.) e. See c.) f. See c.) g. Observed SDM measured by OPTION5 significantly differed between CG (median score of 15) and IG (median score of 35). Perceived SDM measured by SDMQ-9 as well as decisional conflict and decision regret did not significantly differ between CG and IG nor between the 1-week and 3-month time points
Liu et al. (2018, USA)	1. Information and Activation using an evidence-based asthma SDM toolkit 2. Children & adolescents with asthma	Delay asthma exacerbations (e.g., hospitalization) with SDM intervention	Online SDM toolkit with step-by-step instructions including (1) Assessment of asthma control (2) Review of goals and medication preferences (3) Education on asthma medications (4) Inhaler technique (5) Triggers	1) Quantitative: Prospective cohort study with control group (*n* = 746 children & adolescents; *n* = 625 received usual care; *n* = 121 received SDM) 2) Time from first SDM session to asthma exacerbation (defined as either asthma-related hospitalization, emergency department visit or visit with oral steroid prescription) in months	• 14% of youth in IG (vs. 31% in CG) had an asthma exacerbation in the follow-up period • Youth in CG had twice as many oral steroid prescription orders as youth in SDM condition • Median time to exacerbation was 27.65 months in IG vs. 29.35 months in CG (*p* = 0.59) • Kaplan-Meier curves suggested that SDM is associated with significantly lower probability of experiencing exacerbations over time (*p* = 0.005)
Matula et al. (2022, USA)	1) Information, Activation and Collaboration using “iBDecide”: web-based decision aid (DA) tool 2) Adolescents and young adults (up to 21 years) with ulcerative colitis (UC)	Promote SDM among adolescents with UC who are beginning to manage their treatment and medications	(1) Patient Profile: Patient Bio, When to Call Doctor, Preference Settings (2) Tracking a. Nutrition: Nutrition Lists with sections “Safe Choices”, “Triggers” and “Sick Day” b. Medications: Current Medications, Past Medications, Medication Reminders c. Tracker: My Symptoms (3) My Notes (4) Treatment Options a. What is UC? b. What are my treatment options? c. Current Treatment Plan d. Treatment Generator e. Treatment Generator History (5) Appointment Guide a. Notes Section b. Next Appointment including Reminders c. Questions to Doctor d. PUCAI Review e. Symptom Summary f. Nutrition Summary g. Add Next Appointment h. Past Appointments	1) Quantitative: Randomized controlled trial, pilot study (*n* = 16 CG, *n* = 19 IG) 2) Usability of present DA: a. System Usability Scale (SUS; Brooke, 1996) b. 18-item survey adapted by Brinkman et. al, 2013 and Weymiller et al., 2007 Decision outcome: c. Control Preference Scale (Degner et al., 1997) d. Control Preference Scale-post (Brom et al., 2014) e. Decision Conflict Scale (O’Connor, 1995 & 2019) f. Shared Decision Questionnaire (Kriston et al., 2010) g. Usage data	• Usability outcome: Overall participants found iBDecide easy to use and beneficial with a median above 3 on a 5-point Likert scale. Helpfulness of the information and how quick it would be to learn resulted in a median of 5 • Decision Outcome: No decision‐making data are reported as only a few participants reported a decision was made
Moore et al. (2019, USA)	1. Information and Activation using a decision aid in the form of a brochure 2. Adolescents with severe obesity	Facilitate decision-making between HCPs and patient about two treatment options	HCPs use brochure in clinical encounter with the following content: a.) Overview of health problem b.)The treatment decision to be made c.) Two treatment options (intensive lifestyle changes vs. bariatric surgery) d.) Requirements for maximizing success e.) Pictorial risk and benefit comparison of the two options. There are built-in talking points to start the discussion about treatment options	1) Quantitative: Non-controlled posttest design (*n* = 31) 2) Assessment directly after clinical encounter a. Perceived SDM-process: adapted items of CollaboRATE (Barr et al., 2014) b. Understanding of treatment options, self-efficacy in decision-making, decision-making conflict c. Self-developed HCP questionnaire for feasibility and acceptability (Moore et al.,^40^)	a. Patients and families indicated that HCPs made an effort to improve their understanding and to consider and incorporate their preferences and values into the decision-making process (8.6–8.8 out of a possible 9 points) b. 96% knew the benefits and risks of the options. 93% agreed that they received enough support and guidance to make a decision. 89% felt confident about what was the best option for them c. The built-in talking points facilitated the HCPs to start the discussion about treatment options and structured the conversation. They allowed them to guide the family in the spirit of motivational interviewing. HCPs desired additional treatment options in the brochure
Pollak et al. (2020, USA)	1. Information and Activation using the communication intervention „TIC TAC“ 2. Adolescents with various conditions and HCPs	Increasing the participatory behavior of adolescents	Adolescents: Sheet with communication tips, assessment of risk behavior, reflecting on behavior change, encouraging open communication with HCPs HCPs: Summary of risk behaviors of adolescents, tips for motivational interviewing	1) Quantitative: Randomized controlled trial, feedback guide for adolescents and HCPs vs. guide for HCPs only (*n* = 12 HCPs, n = 29 adolescents, *n* = 20 at follow-up) 2) a. Participatory behaviors in audio recordings: Street Active Patient Participation Behaviors coding protocol (Street et al., 2005) b. Perceived empathy of HCPs: CARE (Mercer & Reynolds, 2002) c. Perceived autonomy: William’s Climate Questionnaire (Williams et al., 1996) d. Duration of clinical encounter in minutes e. Behavior changes at follow-up: Rapid Assessment for Adolescents Preventive Services (Salerno, Marshall, & Picken, 2012)	a. Adolescents who received the feedback guide were more likely to demonstrate participatory behaviors, ask more questions (M = 2.8, SD = 5.4 vs. M = 1.9, SD = 3), express more concerns (M = 1.8, SD = 3.1 vs. M = 0.2, SD = 0.4), and be more self-aware (M = 2.1, SD = 23 vs. M = 1.2, SD = 1.8). b. IG rated HCPs more empathetic than CG (M = 4.9, SD = 0.1 vs. M = 4.6, SD = 0.6). c. IG rated HCPs’ support of their autonomy higher (M = 6.4, SD = 0.8 vs. M = 6, SD = 1.3) d. Encounters in IG lasted slightly longer (M = 28.3, SD = 21.5 vs. M = 26.4, SD = 9.7) e. 50% of IG (vs. 40% CG), indicated that they would change their behavior as a result of talking to HCP
Rowe et al. (2018, England)	1. Information and Activation using the decision aid „My Self-Help Tool” 2. Adolescents with self-harm	Support in decision-making about seeking help for self-harm	Web-based personalized decision aid: a.) General information on mood and feelings b.) List of potential sources of support (e.g., parents, HCPs, telephone service) rated by adolescents according to likelihood of use c.) List of statements from which to select the five most important (“I want face-to-face help”, “I don’t want my parents to know about my self-harm”) d.) The five selected statements are again evaluated in general terms for relevance and specifically for each of the self-help options indicated as likely (e.g., discretion more important with friends than with HCPs) e.) Presentation of different self-help options according to individual preferences	1) Mixed-methods quantitative : Randomized controlled trial (IG: *n* = 10; CG: *n* = 13) Qualitative: Thematic analysis (*n* = 23) 2) Assessment before intervention, after intervention, four-week follow-up: Quantitative: a. Feasibility/Acceptance b. Willingness to participate: Stage of Decision-Making Scale (O’Connor, 2000) c. Help-seeking intentions/ experiences: General-Help-Seeking Questionnaire (Wilson et al., 2005) d. Questionnaire on perceived discrimination due to illness (Goodwin et al., 2013) e. Decision-making conflict: Decisional Conflict Scale (DCS; O’Connor, 1995) Qualitative: f. Reasons for use g. Experience with intervention	a. All adolescents in IG stated: to follow the advice of the decision aid; that the decision aid has changed their attitude toward help-seeking behavior; they would recommend the decision aid to others b. no significant differences c. no significant differences d. no significant differences e. no significant differences f. Anonymity, discretion g. Easy and quick to use, increased awareness of sources of support, reduction of shame
Rexwinkel et al. (2021, Netherlands)	1. Information and Activation using the communication aid “3 Good Questions” (3GQ) 2. Children and adolescents with various conditions	Improving the quality of information in consultations between patients and HCPs	Three questions to guide consultation a.) What is it I feel? b.) What can we do about it? c.) What does it mean for me now and later? In addition, information on SDM and children’s right to have a say in form of (1) Brochures for children before consultation with links to videos (2) Card to take in the waiting room (3) Posters and information on digital screens in the waiting room	1) Quantitative: Non-randomized comparison before and after the implementation of 3GQ (*n* = 282) 2) a. Usage b. Perceived participation: SDM-Q-9 (Kriston et al., 2010) c. CollaboRATE (Barr et al., 2014) d. Preference for an active healthcare role: Control Preference Scale (Degner, Sloan, & Venkatesh, 1997)	a. Only 25% with 3GQ (vs. 17% without 3GQ) prepared questions prior to consultation b. 3GQ led to more joint decisions (*p* < 0.001) c. Differences in CollaboRATE not significant d. Most children, regardless of group, prefer a collaborative role in decision-making (>50%)
Simmons et al. (2017a, Australia)	1. Information and Activation using the online decision aid “Youth Depression Decision Aid” 2. Adolescents and young adults (up to 25 years) with depression	Facilitate SDM, decision-making based on individual values and preferences	Website for use by patient and HCP a.) Mood questionnaire: Patient Health Questionnaire (PHQ-9, Kroenke, Spitzer, & Williams, 2001) b.) “What Matters To You” to assess individual needs, values and preferences c.) Treatment options (depending on the severity of PHQ-9 symptomatology) with information and comparison between options d.) „Your Decision”: Known causes of decision conflicts are put up for discussion, participants are asked whether they have enough knowledge, are clear about their values and have enough support to make decisions	1) Quantitative: Uncontrolled cohort study (*n* = 57, *n* = 48 at follow-up). 2) Assessment before the decision, directly after the decision and after 8 weeks: a. Perceived participation: 11-item Shared Decision-Making-Questionnaire (SDM-Q; Simon et al., 2006) b. Satisfaction with decision: Satisfaction with Decision Scale (SWD; Wills & Holmes-Rovner, 2003) c. Decision-making conflict: Decisional Conflict Scale (DCS; O’Connor, 1995) d. Follow-up: Open-ended questions specific to chosen treatment, responses coded for adherence, depression score with PHQ-9 and SWD	a. High level of perceived participation (M = 37.4 of 44, SD = 4.3) b. High level of satisfaction (M = 25.8 of 30, SD = 3.1). c. significant reduction in decision-making conflict between baseline and after the use of decision aid (*p* < 0.001) d. 76% still in original treatment. Depression scores have decreased significantly compared to baseline (M = 2.7 points lower, 95% CI: 1.4–4 points lower). Still satisfied with made decision (M = 25.3, 95% CI: 23.9–26.8)
Simmons et al. (2017b, Australia)	1. Information and Activation using the online decision aid + Peer work “The Choice Project” 2. Adolescents and young adults (up to 25 years) with depression	Facilitate SDM, make informed decision about treatment options	In addition to the Youth Depression Decision Aid (Simmons et al.,^44^), peer workers are provided to assist with the use of the decision aid and after the appointment with HCP. The peer workers are trained peers who have their own experiences with mental illness and recovery	1) Quantitative: Non-randomized comparative study with historical comparison group (*n* = 229) 2) a. Perceived participation: SDM-Q-9 (Kriston et al., 2010) b. Decision-making conflict: DCS (O’Connor, 1995) c. Self-developed satisfaction questionnaire (Rickwood et al., 2017)	a. Perceived SDM higher in IG (*p* = 0.15) b. Significant decrease in both groups from pre to post appointment (*p* < 0.001 for both groups) c. No significant differences with regard to the level of satisfaction
Toupin-April et al. (2020, Canada)	1. Information and Activation using a paper-based prototype of web-based patient decision aid (PDA) called “JIA Option Map” 2. Children/adolescents (8–18 years) with juvenile idiopathic arthritis (JIA)	Help children/adolescents with JIA make informed and preference-based decisions about pain management options	a.) Introduction explaining what JIA is and how it is treated as well as the goal of the present PDA b.) Step 1: Assessment of pain intensity and identification of pain location on body map c.) Step 2: Questions about current treatments, whether they are followed and helpful and whether there is a wish to change or add pain management options d.) Step 3: Values-clarification exercise with 6 questions asking about preferred types of treatment e.) Step 4: Description of 33 treatment options (divided into 6 categories) with information on benefits, risks, and practical aspects based on evidence f.) Step 5: Asks users to make a decision, assessment of motivation and confidence in following their new plan and enabling factors and barriers to following the new plan g.) Step 6: SURE test (Légaré, 2010) to assess how comfortable one feels with the decision	1) Qualitative: Semi-structured individual interviews (*n* = 12 adolescents, *n* = 12 parents, *n* = 11 HCPs) 2) Acceptability testing for present PDA (perceived usefulness, content, format, and future use): Simple descriptive content analysis	(1) 10 adolescents thought that PDA contained appropriate amount of information and appreciated the many treatment options, some novel to them (2) Some parents wished to have more information on complementary medicine, nutrition, and cannabinoids (3) Some adolescents wished to have more information on immediate ways to manage pain and on medication to control disease activity (4) Most participants appreciated the incorporation of scientific evidence, including the visuals showing the probability of benefits, but also said that evidence should be elaborated upon (5) Most participants mentioned that they wished to have some time to review information, followed by discussion on pain management options with HCP (6) All participants agreed that the present PDA is useful for making decisions about pain management in JIA as it is increasing their knowledge of options, helping with awareness of preferences, helping be in control of their own decisions, and reassuring that they are choosing the best options
Walker et al. (2017, USA)	1. Information and Activation using individual training/coaching “Achieve My Plan” (AMP) 2. Adolescents with mental health problems	Increase satisfaction, active engagement, and self-determined participation in team meetings	Three one-on-one coaching sessions: trained coaches, preparation for team meetings a.) Identifying strengths and long-term goals (personally meaningful and motivating), develop action steps for sharing goals with team. b.) Set agenda for team meeting, what adolescents want to say and when (e.g., ask team members for support, describe own role in action steps) c.) Preparation for active participation in group meeting, Coaches help adolescents to anticipate conflicts that might arise during the meeting and discuss strategies for dealing with these situations, what to do when adolescent gets angry or anxious and does not remember what he/she wanted to say Two booster sessions: between preparatory sessions (e.g., review progress, repeat important content) Team meeting: includes e.g., adolescent family members, HCPs/TPs working with family, family social support network persons. Coach models behaviors that encourage adolescent participation (e.g., reminds team to talk directly to adolescents and not about them)	1) Quantitative: Randomized controlled trial (n = 55) 2) Assessment (except for videotaping) at baseline (T1), immediately after the team meeting (T2), and 5 weeks afterward (T3) a. Perceived participation in team-based planning: Youth Participation in Planning Scale (YPP; Walker & Powers, 2007) b. Perceived relationship with HCPs/TPs: Working Alliance Inventory (WAI; Horvath & Greenberg, 1989) c. Empowerment/ Support of children: Youth Empowerment Scale – Mental Health (YES; Walker et al., 2010) d. Video recording of the group session: team interaction, juvenile contributions to discussions, speaking time, assessed by trained observers	a. Between T1 and T2, as well as between T1 and T3, the main effect for the intervention was significant (*p* < 0.01) b. Adolescents in IG rated teamwork significantly better than CG (*p* < 0.01) c. No significant differences d. Mean differences in favor of IG, but differences were not significant
Wysocki et al. (2018, USA)	1. Information and Activation using two internet-based, multimedia decision aids (DA) called “My Decision: Getting Insulin” and “My Decision: Checking Blood Sugar” 2. Children/Adolescents (11–17 years) with Type 1 Diabetes (T1D) as part of an adolescent/caregiver dyad	Facilitate decision between using either an insulin pump or continuous glucose monitoring (CGM) and treatment as usual regarding T1D, improve decision quality, patients’ knowledge of treatment options, patient engagement, satisfaction and health outcomes since DAs should enhance SDM	Both DAs include the same structural components: a.) Welcome Tour: A 4 min animated and narrated introduction to the DA features; b.) My Quiz: A brief self-assessment of one’s readiness for adding the insulin pump or CGM to their care; c.) Pumps and Shots (or CGM and Home Glucose Meter) at a Glance: A comparison of the pros and cons of each device; d.) Everyday Tasks and Responsibilities: Teens telling in their own words what they have to do each day to use the insulin pump or CGM to get the most out of it; e.) How an Insulin Pump (or CGM) Works: A brief illustrated summary of how the devices work and what’s required to use them properly; f.) What’s it Like to Have an Insulin Pump (or CGM)?: Teens telling in their own words what it is/was like to use an insulin pump (or CGM); g.) 5 Things to Know about Pumps (or CGM): The five most important facts that teens say other teens should know about each device before deciding to get one; h.) 5 Things to Know about Shots (or Home Glucose Meters): The five most important facts that teens say other teens should know about staying with the devices they are using now; i.) Talking With Your Family: Suggestions from teens and parents about effective and ineffective ways to make a decision about adopting an insulin pump or CGM; j.) Frequently Asked Questions: Teens and parents suggested these questions that they wish they could have answered before starting on an insulin pump or CGM; k.) What Can Go Wrong?: Each device can be a problem if certain things go wrong. What are some of the problems and how can teens prevent them from happening?; l.) My Progress: This circular symbol illustrates how much or how little of the DA website the logged-in user has opened; m.) Decision Slider: A user can move the decision slider on each page to show how much a given section of the website caused them to shift their opinion toward or away from the insulin pump or CGM; n.) Glossary: A searchable list of T1D-related technical definitions stated in plain language.	1) Quantitative: Randomized controlled trial (*n* = 133 adolescent/caregiver dyads) 2) Electronically verified indices of various dimensions of participants’ DA use during decision-making process in 3-month study period as outcome measures in planned regression models with demographic data as possible predictor variables a. Categorization of participants as DA user or nonuser b. Frequency of DA logins c. Duration of DA use (for a given DA use event and total duration between first login and last logout) d. Percentage of DA content viewed e. Use of decision slider indicative of greater likelihood of either accepting or declining insulin pump or CGM	Extent of adolescent and caregiver DA use was significantly and positively associated for all DA use variables (at least: *p* < 0.006) a. For caregivers and adolescents, no individual demographic predictor variable was associated significantly with membership in User/Nonuser groups; neither health literacy nor health numeracy scores emerged as significant predictors for group membership b. For caregivers, no individual demographic predictor variable was associated significantly with DA login frequency. For adolescents, gender (*p* = 0.05) and REALM scores (Rapid Estimate of Adolescent Literacy in Medicine; *p* < 0.002) emerged as significant demographic predictors for DA login frequency, indicating that males with lower health literacy scores demonstrated fewer DA logins c. For caregivers and adolescents, no individual demographic predictor variable was associated significantly with duration of DA use d. For caregivers and adolescents, no individual demographic predictor variable was associated significantly with percentage of DA content viewed e. Among adolescents, a narrow minority (46.8%) tended toward adoption of either insulin pump or GCM instead of treatment as usual; among adolescents, a narrow majority (55.3%) tended toward these options

*HCPs* Healthcare Professionals, *IG* Intervention group, *CG* Control group, *PEP* Patient Engagement Project, *SDM* Shared Decision-Making, *TP* Therapist.

^a^Adapted by the classification by Grande et al.^30^

^b^Reference list of instruments see Supplementary Material S3.

Of the 21 included studies, 8 were conducted in the United States, 3 studies each are from Canada and the United Kingdom, 2 studies originate from Australia and 1 study each is from Sweden, Egypt, Norway, Japan, and the Netherlands. All studies were published in 2017 or later.

Most of the studies (*n* = 13) used only quantitative methods to evaluate the intervention, while 3 studies reported only qualitative data. The remaining 5 have a mixed study design, using both quantitative and qualitative methods. Of the quantitative (and mixed) studies, 7 reported randomized controlled trials. The sample sizes ranged from *n* = 5 to *n* = 746 participants.

Of the 21 interventions, 10 were aimed at both children and adolescents, 6 studies were aimed at adolescents only, and 3 studies aimed at children only. Three studies additionally included young adults (up to 21 years/25 years). One study each focused on adolescents and HCPs and one on children and HCPs. The children and adolescents included in the studies had different conditions. Eleven interventions were specifically designed and used for somatic conditions such as diabetes, cancer, or asthma, while 6 interventions focused on mental health conditions such as depression, anxiety, or self-harm. In 3 studies, no specific health condition was targeted because the intervention was intended to be applied across different conditions. One study examined decision-making processes during dental examinations in healthy children.

The quality of the included studies is heterogeneous with some limitations, but can overall be deemed as moderate to good using the MMAT. A detailed overview of the MMAT quality assessment for each study is provided in Table S1 (Supplementary Material).

### Aims and didactic strategies

The majority of studies (*n* = 15)^33–47^ aimed *to inform or educate* patients about their patient’s health condition, treatment options, principles, and behavior patterns of SDM either alone (*n* = 3) or as a base for further intervention steps (*n* = 12). Further, interventions (*n* = 6) aim *to support active involvement of patients in discussions* with HCPs regarding further treatment^37,40,43–45,48^ or to involve patients’ treatment-related questions, wishes, or preferences *indirectly* (e.g., by question lists) without a direct conversation between patients and HCPs.^35,41,42,47,49–51^ In terms of goal setting and engaging in decision-making, interventions employed a *decision aid* to assist patients in planning upcoming discussions with their HCPs (*n* = 8).^34,37,39,46,47,51–53^ Eleven interventions combined two aims.^34,35,39–46,51^ Two interventions pursued more than two aims.^37,47^ Overall, most interventions employed strategies to enhance information and activation. Few interventions additionally aimed to engage children and adolescents by explicitly enhancing collaboration.

Thirteen studies used d*igital interactive applications*, available on either a web-based platform or on a mobile device.^33,34,36,38,39,42,44–47,49,50,52^ Twelve interventions used t*reatment protocols* and *preparing guiding questions and decision plans* (*n* = 12),^34,37–39,43–48,51,52^ which patients should keep and prepare. *Implementation of surveys or quizzes* (*n* = 8) concerning patients’ condition or their knowledge on their disease, treatment alternatives, or decision-making behavior generally were used to increase patients’ interest and involvement in the treatment process.^34–36,41,44–47^ Four studies included v*isual aids* to improve SDM processes (*n* = 4), for instance, booklets containing information on SDM^34,40,43,48^ A m*ulti-day training and education course* with the aim to promote SDM practices in pediatric healthcare settings for HCPs, patients, and their parents was used in one study.^53^

In 12 studies, multi-component interventions incorporated two or more of the aforementioned didactic strategies,^34–36,38,39,43–48,52^ For instance, in all seven studies employing digital decision aid tools,^34,39,44–47,52^ the tool was presented as a *digital interactive application* and provided sections for *treatment protocols, guiding questions and decision plans* as well as *questionnaires or quizzes* within the digital space.

### Intervention effectiveness

The statistical methods, sample sizes, and measurement tools differ across studies. We summarized the results of effectiveness based on the outcome variables.

#### Feasibility

Ten studies assessed the feasibility, usability, and/or acceptability of the applied intervention as a specific outcome variable. Most studies found that the tools to be comprehensible, clear, and easy to use.^34,36,37,39,42,51^ Few studies reported negative findings or barriers of use, such as the decision-making intervention taking up too much time.^48,51^ Technical problems were also identified as a barrier for usage.^52^ The communication device used by Rexwinkel^43^ showed low usage rates with only 25% of participants who used the device having prepared questions in advance to consultation with their HCP (in contrast to 17% of those who did not use it) as planned in the program.^43^ Additional aspects reported to be relevant for usability and acceptability included the lack of individualization of information, suboptimal timing of information delivery, and the limited opportunity for direct communication with the HCP.^46^

#### Patient-HCP relationship and therapy adherence

Seven studies investigated the effect of the applied decision-making intervention on patient-HCP relationship and adherence measures. Carlsson et al. investigated the impact of a digital interactive communication tool and found that children were included in communication with the HCP and recognized as a person.^49^ Positive changes in interaction ratings were also reported regarding teamwork with HCPs, the family support network,^53^ and empathy of HCPs.^41^ In contrast, in the study by Langer et al., patients of the intervention group (IG) reported significantly lower therapeutic alliance scores directly following the treatment planning session as part of the SDM intervention (*p* = 0.017). HCPs also reported lower therapeutic alliance scores following treatment planning (*p* = 0.03), but not at mid- or post-treatment assessment.^37^

Therapy adherence was explicitly assessed in three studies: El Miedany et al. found that 88% of IG patients who used an interactive decision aid tool (compared to 71% of patients of the control group (CG)) adhered to their medical therapy (*p* < 0.01).^33^ Simmons et al. reported that 76% of their patient cohort who had used an online decision aid tool were still in their original treatment for depression at follow-up.^44^ Lipstein et al.^48^ found no significant differences in medication adherence between IG and CG patients.^48^

#### Decisional conflict

Eight studies investigated changes in perceived decisional conflict induced by the applied decision-making interventions. Significant reduction of decisional conflict in the IG compared to CGs was reported.^37,44,51^ However, these results could not be demonstrated either at follow-up^44^ nor for specific subgroups of patients with mental disorders.^37^ Other studies found no significant differences in decisional conflict between IG and CG patients^35,42,48^ or did not provide enough data to draw certain conclusions.^39^

#### Health-related knowledge

Four studies explicitly investigated the amount of knowledge conveyed by the decision-making interventions. Qualitative findings suggest increased awareness of social support sources.^42^ One study reported high levels of knowledge about the risks and benefits of treatment after using a patient decision aid.^40^ Moreover, greater condition-specific knowledge was observed in IG compared to CG^31^ or in pre-post comparisons.^44^

#### Participation in decision-making processes

Twelve studies explicitly investigated the direct effect of the applied decision-making intervention on the level of patient participation in decision-making processes. Participants of the IG experienced higher levels of involvement in the decision-making or treatment-planning processes.^33,37,43–45,53^ It has been reported that HCPs showed more interaction, more proactive communication, and more effort to involve patients.^40,50^ More proactive communication (e.g., asking questions, expressing concerns, non-verbal participation) has also been reported in children and adolescents.^41^ Several studies reported no positive effects or differences in IG compared to CG in terms of speaking time,^50–53^ decision self-efficacy,^37^ duration of clinical encounter,^41^ willingness to participate,^42^ and empowerment.^53^ Lipstein et al.^48^ found significant differences between IG and CG patients in observed SDM, but not in subjectively perceived SDM.^48^ The study by Matula et al.^39^ did not provide enough data to draw any conclusions about SDM-related outcomes.^41^

#### Clinical outcome measures

Five studies investigated the effects of the applied intervention on clinical outcome measures. Positive effects were reported for the frequency of asthma exacerbation (asthma attacks, clinic visits, and oral steroid prescription) in asthma patients.^38^ Lipstein et al.^48^ found no positive effects on disease activity scores for pediatric inflammatory bowel disease.^48^ Regarding interventions for children and adolescents with mental disorders, studies show inconsistent findings: Simmons^44^ reported a significant decrease in depression scores compared to baseline,^44^ whereas other studies found no positive effects of the applied decision-making intervention on mental health outcomes in IG patients compared to CG.^35,37^

#### Other outcome parameters

Qualitative results suggest increased self-reflexivity, improved emotional state, and ability to weigh pros and cons of relevant issues after the application of a decision-making intervention.^52^ Moreover, significantly higher levels of quality of life were reported compared to CG.^33^ Studies investigating satisfaction with decisions found higher satisfaction among HCPs compared to CG^37^ and high levels of satisfaction with the decision following the intervention.^44,45^ However, Simmons et al. found no differences in satisfaction compared to a historical comparison group,^45^ and Lipstein et al.^48^ did not identify differences in quality of life between IG and CG.^48^

## Discussion

Decision-making in the context of pediatric healthcare has received increasing attention in recent years. While other reviews on decision-making interventions focused on parents as the target group,^1^ only partially included interventions in the pediatric context^27^ or did not include interventions that focus on children,^26^ our review focused primarily on interventions for children and adolescents that aim at increasing participation in healthcare decisions. Moreover, we only included studies that reported results on the feasibility or effectiveness of the interventions based on original data.

In total, we identified 21 publications including 20 different interventions to increase the participation of children and/or adolescents in decision-making. Most of the interventions aimed at information and activation. Only few interventions also explicitly focused on collaboration during an encounter.^30^ A wide range of didactic strategies were used to convey the content of the interventions: digital interactive applications, guiding questions or plans, questionnaires and quizzes, visual aids, or trainings/educational sessions.

Fourteen of the 21 studies included in our review were published after 2019, indicating a trend toward the development and evaluation of interventions targeting children and adolescents. The interventions show high feasibility and acceptability rates among participants and appear to be helpful. In some studies, technical or other usage barriers were reported, information was perceived as too much (overloaded decision-aid tools) or to little (poorly understandable decision-aid tools). These aspects should be considered when developing interventions. One promising way to address potential barriers may be to already include pediatric patients in the developmental process of an intervention.^54^

The results suggest that the interventions have contributed to increasing the level of patient participation in decision-making processes. Most studies identified a positive effect of interventions on the HCP-patient relationship, therapy adherence, and health knowledge. However, few studies did not report improvements in specific outcome parameters. Inconsistent results were found for the impact of interventions on decisional conflict or clinical outcomes. The findings are similar to those of previous reviews mainly including interventions targeting at parents. These also identified rather positive effects of the interventions.^1,55^ However, methodological shortcomings of the included studies are discussed and interventions were mostly aimed at parents.^1,55^

Similarly, the studies in our scoping reviews are very heterogeneous in terms of the statistical methods, sample sizes, measurement tools, and interpretations of whether and how an intervention could improve patients’ involvement in decision-making processes, making it difficult to synthesize and compare the results and draw overarching evidence about effectiveness. Further research is needed to understand the processes and to identify factors that influence the effects of decision-making interventions.

To provide an overview of existing interventions, we included most types of patients and diseases. Differences between diagnostic groups (e.g., mental vs. physical illness) could be investigated in the future. Boland et al. identify various barriers and facilitators for SDM on the part of patients and parents (e.g., health status, emotional state, socioeconomic status, or language) as well as professionals (e.g. SDM skills, specialty, role).^56^ In the future, it could be interesting to see how the identified interventions can be adapted with regard to these barriers and how effective they are.

### Limitations

A limitation of this review is that abstract and title screening was only partly conducted by two independent reviewers. We may have missed studies at this review stage. However, we conducted an extensive search of citations and references by two reviewers to minimize the possibility of missing relevant literature. Due to our aim of providing a broad overview of existing interventions and their feasibility and effectiveness, the depth of detail of the analysis was limited. Moreover, we did not include interventions focusing on hypothetical decisions, non-medical decisions (e.g., school), decisions about pregnancy, peri- or neonatal care, advanced care planning, decisions about participation in research, or self-management interventions without a focus on decision-making. Three studies also included young adult patients, who may not be comparable to children or adolescents, for example, because of different legal situations regarding own decision-making.

Another limitation of this review is the quality of the underlying data. In our review, 7 of the 21 included studies followed a randomized controlled study design. The MMAT revealed moderate to good quality within the applied study design. However, many studies have methodological shortcomings such as small sample size or lack of CG. Moreover, there seems to be a lack of standardized outcome measures for (shared) decision-making in the context of children’s health. Therefore, different study designs and different outcome measures hinder the overall estimation of the effects of decision-making interventions.

## Conclusions

This scoping review provides an overview of existing interventions for children and adolescents to increase participation in decision-making in the healthcare context or to facilitate SDM. Most interventions provided information and encourage children and adolescents to participate in the decision process, e.g., digital information tools and digital decision aids. Few interventions additionally used collaborative approaches. The results support the feasibility of the interventions and indicate preliminary positive effects of the interventions on certain outcome parameters such as participation, adherence, or health-related knowledge. However, the underlying database does not allow for full conclusions. Further high-quality studies investigating the effects are needed. Standardized outcome measures and further randomized controlled trials should be used to increase the generalizability of the findings.

## Supplementary information

Supplementary Material S1

Supplementary Material S2

Supplementary Material S3

Table S1

Table S2

## Data Availability

All relevant data are presented within the manuscript and supplementary material.
