# Supplementary material for: Decision-making for children and adolescents: a scoping review of interventions increasing participation in decision-making
Source: Pediatr Res. 2024 Oct 6;97(6):1840–54. doi: 10.1038/s41390-024-03509-5 (PMC12122360; doi:10.1038/s41390-024-03509-5)
Supplement: Supplementary file 2 — Supplementary Material S2 [file 41390_2024_3509_MOESM2_ESM.pdf]

Facilitating shared decision-making in pediatric health care – scoping review on decision-making interventions focusing on children and adolescents

Supplementary Material S2.

Search Strategy

#	Query PubMed	Results 23.09.21
#1	"Decision Making"[MESH] OR „decision making“[Title/Abstract] OR „shared decison making“[Title/Abstract]	333.770
#2	Informed[Title/Abstract] OR shared[Title/Abstract] OR voicing[Title/Abstract] OR participat*[Title/Abstract] OR interactive[Title/Abstract] OR tool*[Title/Abstract] OR aid[Title/Abstract] OR aids[Title/Abstract] OR technique*[Title/Abstract] OR instrument[Title/Abstract] OR support[Title/Abstract]	4.407.373
#3	Decide[Title/Abstract] OR decision*[Title/Abstract] OR voice*[Title/Abstract] OR choice*[Title/Abstract] OR communication[Title/Abstract] OR conversation[Title/Abstract]	1.065.325
#4	#2 AND #3	358.514
#5	#1 OR #4	602.438
#6	"Patient Participation"[Mesh] OR „patient involvement“[Title/Abstract] OR „patient participation“[Title/Abstract] OR „patient engagement“[Title/Abstract] OR „patient satisfaction“[Title/Abstract] OR „patient preference*“[Title/Abstract] OR „patient centered“[Title/Abstract] OR „patient empowerment“[Title/Abstract] OR „patient activation“[Title/Abstract] OR „patient support“[Title/Abstract]	103.890
#7	"Pediatrics"[Mesh] OR child*[Title/Abstract] OR adolescent*[Title/Abstract] OR pediatric*[Title/Abstract] OR paediatric*[Title/Abstract] OR girl[Title/Abstract] OR boy[Title/Abstract] OR youth[Title/Abstract] OR juvenile[Title/Abstract] OR teen*[Title/Abstract]	2.043.602
#8	"Health Services"[Mesh] OR „patient care“[Title/Abstract] OR „child care“[Title/Abstract] OR „child health services“[Title/Abstract] OR „adolescent health services“[Title/Abstract]	2.329.837
#9	#5 AND #6 AND #7 AND #8	1164

S	Query CINAHL	Results 23.09.21
S1	(MH "Decision Making, Shared“) OR (MH "Decision Making, Patient“) OR (MH "Decision Support Systems“) OR TI (decision making OR shared decision making) OR AB (decision making OR shared decision making)	87.228
S2	TI (informed OR shared OR voicing OR participat* OR interactive OR tool* OR aid OR aids OR technique* OR instrument OR support) OR AB (informed OR shared OR voicing OR participat* OR interactive OR tool* OR aid OR aids OR technique* OR instrument OR support)	1.127.223
S3	TI (decide OR decision* OR voice* OR choice* OR communication OR conversation) OR AB (decide OR decision OR voice* OR choice* OR communication OR conversation)	389.685
S4	S2 AND S3	151.581
S5	S1 OR S4	199.409
S6	(MH "Consumer Participation“) OR (MH "Physician-Patient Relations“) OR (MH "Nurse-Patient Relations“)	82.017
S7	TI (consumer participation OR patient participation OR patient involvement OR patient engagement OR patient satisfaction OR patient preference* OR patient centered OR patient empowerment OR patient activation OR patient support) OR AB (consumer participation OR patient participation OR patient involvement OR patient engagement OR patient satisfaction OR patient preference* OR patient centered OR patient empowerment OR patient activation OR patient support)	102.885
S8	S6 OR S7	146.347
S9	(MH "Pediatrics“) OR (MH "Child“)	502.131
S10	TI (Pediatric* OR paediatric* OR Child* OR adolescent* OR girl OR boy OR youth OR juvenile OR teen*) OR AB (Pediatric* OR paediatric* OR Child* OR adolescent* OR girl OR boy OR youth OR juvenile OR teen*)	748.145
S11	S9 OR S10	904.425
S12	(MH "Health Services“) OR (MH "Child Health Services“) OR (MH "Child Health“)	38.974
S13	TI (Health Services OR child health OR adolescent health OR child health services OR adolescent health services OR patient care OR child care OR pediatric care) OR AB (Health Services OR child health OR adolescent health OR child health services OR adolescent health services OR patient care OR child care OR pediatric care)	385.738
S14	S12 OR S13	411.604
S15	S5 AND S8 AND S11 AND S14	758

#	Query PsycINFO	Results 23.09.21
#1	"decision making".sh. or "decision making".ti,ab. or "shared decision making".ti,ab.	122.047
#2	(Informed or shared or voicing or participat* or interactive or tool* or aid or aids or technique* or instrument or support).ti,ab.	1.248.568
#3	(Decide or decision* or voice* or choice* or communication or conversation).ti,ab.	554.847
#4	2 and 3	188.719
#5	1 or 4	266.585
#6	"Client participation".sh. or "client participation".ti,ab. or "patient involvement".ti,ab. or "patient participation".ti,ab. or "patient engagement".ti,ab. or "patient satisfaction".ti,ab. or "patient preference*".ti,ab. or "patient centered".ti,ab. or "patient empowerment".ti,ab. or "patient activation".ti,ab. or "patient support".ti,ab.	15.729
#7	Pediatrics.sh. or child*.ti,ab. or adolescent*.ti,ab. or pediatric*.ti,ab. or paediatric*.ti,ab. or girl.ti,ab. or boy.ti,ab. or youth.ti,ab. or juvenile.ti,ab. or teen*.ti,ab.	943.234
#8	"health care services".sh. or "health care service*".ti,ab. or "health service".ti,ab. or "patient care".ti,ab. or "child care".ti,ab. or "child health service*".ti,ab. or "adolescent health service*".ti,ab.	82.331
#9	5 and 6 and 7 and 8	45
