# Supplementary material for: Decision-making for children and adolescents: a scoping review of interventions increasing participation in decision-making
Source: Pediatr Res. 2024 Oct 6;97(6):1840–54. doi: 10.1038/s41390-024-03509-5 (PMC12122360; doi:10.1038/s41390-024-03509-5)
Supplement: Supplementary file 3 — Supplementary Material S3 [file 41390_2024_3509_MOESM3_ESM.pdf]

Supplementary Material S3.

Reference list of measurement instruments in Table 2

Paul J. Barr, Alistair James O'Malley, Maka Tsulukidze, Michael R. Gionfriddo, Victor Montori, Glyn Elwyn, The psychometric properties of Observer OPTION5, an observer measure of shared decision making, Patient Education and Counseling, Volume 98, Issue 8, 2015, Pages 970-976, ISSN 0738-3991, https://doi.org/10.1016/j.pec.2015.04.010.

William B. Brinkman, Jessica Hartl Majcher, Lauren M. Poling, Gaoyan Shi, Mike Zender, Heidi Sucharew, Maria T. Britto, Jeffery N. Epstein, Shared decision-making to improve attention-deficit hyperactivity disorder care, Patient Education and Counseling, Volume 93, Issue 1, 2013, Pages 95-101, ISSN 0738-3991, https://doi.org/10.1016/j.pec.2013.04.009.

Brooke J. SUS: A "quickdirty" usability scale. Jordan PW, Thomas B, Weerdmeester BA. & McClelland IL (Eds), Usability evaluation in industry. London: Taylor & Francis. pp 1996:189-194. [doi: 10.1201/9781498710411-35]

Levente Kriston, Isabelle Scholl, Lars Hölzel, Daniela Simon, Andreas Loh, Martin Härter, The 9-item Shared Decision Making Questionnaire (SDM-Q-9). Development and psychometric properties in a primary care sample, Patient Education and Counseling, Volume 80, Issue 1, 2010, Pages 94-99, ISSN 0738-3991, https://doi.org/10.1016/j.pec.2009.09.034.

Shepanski, Melissa A.; Markowitz, Jonathan E.; Mamula, Petar; Hurd, Linda B.; Baldassano, Robert N.. Is an Abbreviated Pediatric Crohn's Disease Activity Index Better Than the Original?. Journal of Pediatric Gastroenterology and Nutrition 39(1):p 68-72, July 2004.

Steiner, J. F., Koepsell, T. D., Fihn, S. D., & Inui, T. S. (1988). A General Method of Compliance Assessment Using Centralized Pharmacy Records: Description and Validation. Medical Care, 26(8), 814–823. http://www.jstor.org/stable/3765465

Dan Turner, Anthony R. Otley, David Mack, Jeffrey Hyams, J. de Bruijne, Krista Uusoue, Thomas D. Walters, Mary Zachos, Petar Mamula, Dorcas E. Beaton, A. Hillary Steinhart, Anne M. Griffiths, Development, Validation, and Evaluation of a Pediatric Ulcerative Colitis Activity Index: A Prospective Multicenter Study, Gastroenterology, Volume 133, Issue 2, 2007, Pages 423-432, ISSN 0016-5085, https://doi.org/10.1053/j.gastro.2007.05.029.

James W. Varni, Tasha M. Burwinkle, Michael Seid, Douglas Skarr, The PedsQL™* 4.0 as a Pediatric Population Health Measure: Feasibility, Reliability, and Validity, Ambulatory Pediatrics, Volume 3, Issue 6, 2003, Pages 329-341, ISSN 1530-1567, https://doi.org/10.1367/1539-4409(2003)003<0329:TPAAPP>2.0.CO;2.

Weymiller AJ, Montori VM, Jones LA, et al. Helping Patients With Type 2 Diabetes Mellitus Make Treatment Decisions: Statin Choice Randomized Trial. Arch Intern Med. 2007;167(10):1076–1082. doi:10.1001/archinte.167.10.1076
