# Supplementary material for: Decision-making for children and adolescents: a scoping review of interventions increasing participation in decision-making
Source: Pediatr Res. 2024 Oct 6;97(6):1840–54. doi: 10.1038/s41390-024-03509-5 (PMC12122360; doi:10.1038/s41390-024-03509-5)
Supplement: Supplementary file 4 — Table S1 [file 41390_2024_3509_MOESM4_ESM.pdf]

Table S1. Quality assessment of included studies using Mixed Methods Appraisal Tool (version 2009 [26])

References	Methodological approach	Appraisal of quality			
	1. Qualitative	1.1 Are the sources of qualitative data relevant to address the research question?	1.2. Is the process for analyzing qualitative data relevant to address the research question (objective)?	1.3. Is appropriate consideration given to how findings relate to the context, e.g., the setting, in which the data were collected?	1.4. Is appropriate consideration given to how findings relate to researchers' influence, e.g., through their interactions with participants?
Carlsson et al., 2021		Yes	Yes	Yes	Yes
Eldbrooke-Childs, 2019		Yes	Yes	Yes	Yes
Gilljam et al., 2020		Yes	Yes	Yes	Yes
Ho et al., 2021		Yes	Yes	Yes	Yes
Hulin et al., 2017		Yes	Yes	Yes	No
Iio et al., 2022		Yes	Yes	Yes	No
Rowe et al., 2018		Yes	Yes	Yes	Yes
Toupin-April et al., 2020		Yes	Yes	Yes	No
	2. Quantitative randomized controlled	2.1. Is there a clear description of the randomization (or an appropriate sequence generation)?	2.2. Is there a clear description of the allocation concealment (or blinding when applicable)?	2.3. Are there complete outcome data (80% or above)?	2.4. Is there low withdrawal/drop-out (below 20%)?
El Miedany et al., 2019		Yes	No	Yes	Yes
Langer et al., 2022		Yes	No	Yes	Yes
Matula et al. 2022		Yes	Yes	No	Yes
Pollak et al., 2020		Yes	No	Unclear	No
Rowe et al., 2018		Yes	Yes	Yes	Yes
Walker et al., 2017		Yes	Unclear	Yes	Yes
Wysocki et al., 2018		Yes	No	Unclear	Unclear
	3. Quantitative non-randomized	3.1. Are participants (organizations) recruited in a way that minimizes selection bias?	3.2. Are measurements appropriate regarding the exposure/ intervention and outcomes?	3.3. In the groups being compared are the participants comparable, or do researchers take into account	3.4. Are there complete outcome data (80% or above), and, when applicable, an acceptable response rate (60% or above), or an

				(control for) the difference between these groups?	acceptable follow-up rate for cohort studies (depending on the duration of follow-up)?
Gilljam et al., 2020		Yes	Yes	Yes	Yes
Hulin et al., 2017		Yes	Yes	Yes	Yes
Lawson et al., 2020		Yes	Yes	Yes	Yes
Lipstein et al., 2021		Yes	Yes	Yes	Yes
Rexwinkel et al., 2021		Yes	Yes	Yes	Yes
Simmons et al., 2017a		Yes	Yes	Yes	Yes
Simmons et al., 2017b		Yes	Yes	Yes	Yes
	4. Quantitative descriptive	4.1. Is the sampling strategy relevant to address the quantitative research question?	4.2. Is the sample representative of the population understudy?	4.3. Are measurements appropriate (clear origin, or validity known, or standard instrument)?	4.4. Is there an acceptable response rate (60% or above)?
Ho et al., 2021		Yes	Yes	Yes	Unclear
Iio et al., 2022		Yes	Yes	No	Yes
Liu et al., 2018		Yes	Yes	No	Yes
Moore et al., 2019		Yes	Yes	Yes	Yes
	5. Mixed	5.1. Is the mixed methods research design relevant to address the qualitative and quantitative research questions (or objectives), or the qualitative and quantitative aspects of the mixed methods question (or objective)?	5.2. Is the integration of qualitative and quantitative data (or results*) relevant to address the research question (objective)?	5.3. Is appropriate consideration given to the limitations associated with this integration, e.g., the divergence of qualitative and quantitative data (or results*) in a triangulation design?	*Criteria for the qualitative component (1.1 to 1.4), and appropriate criteria for the quantitative component (2.1 to 2.4, or 3.1 to 3.4, or 4.1 to 4.4), must be also applied.*
Gilljam et al., 2020		Yes	Yes	Yes	
Ho et al., 2021		Yes	Yes	No	
Hulin et al., 2017		Yes	Yes	No	
Iio et al., 2022		Yes	Yes	No	
Rowe et al., 2018		Yes	Yes	Yes	
