# Supplementary material for: Decision-making for children and adolescents: a scoping review of interventions increasing participation in decision-making
Source: Pediatr Res. 2024 Oct 6;97(6):1840–54. doi: 10.1038/s41390-024-03509-5 (PMC12122360; doi:10.1038/s41390-024-03509-5)
Supplement: Supplementary file 5 — Table S2 [file 41390_2024_3509_MOESM5_ESM.pdf]

Table S2. Reasons for exclusion after fulltext screening

Author (Year, Country)	Reason for exclusion
Brand McCarthy et al. (2021, USA)	Not feasibility or evaluation data
Daraiseh et al. (2022, USA)	No data reported
Harman et al. (2019, USA)	Focus on healthcare professionals
Hogue et al. (2016, USA)	Not feasibility or evaluation data
Lewis. et al. (1991, USA)	No focus on participation in decision-making
Nicholas et al. (2007, Canada)	No focus on participation in decision-making
Simmons et al. (2018)	No focus on participation in decision-making
Sleath et al. (2018, USA)	No focus on participation in decision-making
Sleath et al (2019, USA)	No focus on participation in decision-making
Stålberg et al. (2019, Sweden)	Not feasibility or evaluation data
Tieffenberg et al. (2000, Argentina)	No focus on participation in decision-making
Ngadimon et al. (2017)	No focus on participation in decision-making
Parikh et al. (2021)	No focus on participation in decision-making
Clark et al. (2000)	Focus on healthcare professionals
Khan et al. (2018)	Focus on parents
Kouo et al. (2021)	No focus on participation in decision-making
Kebbe et al. (2020)	Not feasibility or evaluation data
Christenson et al. (2010)	Not feasibility or evaluation data
Jones & Huggins (2012)	Not feasibility or evaluation data
Lyon et al. (2009)	Advanced care planning
Mesko & Beoglos Eliades (2016)	No focus on participation in decision-making
Nkoy et al. (2021)	No focus on participation in decision-making
Newes-Adeyi et al. (2003)	No focus on participation in decision-making
Bovero et al. (2018)	Not feasibility or evaluation data
Rao-Gupta et al. (2017)	No focus on participation in decision-making
Barfield at al. (2010)	No focus on participation in decision-making
Mende et al. (2017)	No original article
Pembroke et al. (2021)	Not feasibility or evaluation data
Downing et al. (2017)	No focus on participation in decision-making
Fiks et al. (2014)	Not feasibility or evaluation data
Goske et al. (2004)	Not feasibility or evaluation data
Haldar et al. (2021)	Not feasibility or evaluation data
Hannon et al. (2018)	Not feasibility or evaluation data
Martyn et al. (2013)	No focus on participation in decision-making
Pavitt et al. (2021)	Focus on healthcare professionals
Santana et al. (2015)	Focus on adults
Silva Santos et al. (2020)	No focus on participation in decision-making
Snaman et al. (2021)	Focus on adults
Taylor et al. (2018)	No sufficient description of intervention
Valenzuela et al. (2014)	No focus on participation in decision-making
Williams et al. (2015)	No focus on participation in decision-making
Knight et al.	No focus on participation in decision-making
Iio et al. (2020)	Not feasibility or evaluation data
Van Bragt et al. (2014)	No focus on participation in decision-making
